# Influence of Commercial Protease and Drying Process on Antioxidant and Physicochemical Properties of Chicken Breast Protein Hydrolysates

**DOI:** 10.3390/foods10122994

**Published:** 2021-12-04

**Authors:** Phatthawin Setthaya, Sanchai Jaturasitha, Sunantha Ketnawa, Thanongsak Chaiyaso, Kenji Sato, Rawiwan Wongpoomchai

**Affiliations:** 1 Department of Biochemistry, Faculty of Medicine, Chiang Mai University, Chiang Mai 50200, Thailand; phatthawin.l@cmu.ac.th (P.S.); sunantha.k@cmu.ac.th (S.K.); 2 Department of Animal and Aquatic Sciences, Faculty of Agriculture, Chiang Mai University, Chiang Mai 50200, Thailand; 3 Science and Technology Research Institute, Chiang Mai University, Chiang Mai 50200, Thailand; ja.sanchai@gmail.com; 4 Division of Biotechnology, Faculty of Agro-Industry, Chiang Mai University, Chiang Mai 50100, Thailand; tachaiyaso@hotmail.com; 5 Division of Applied Biosciences, Graduate School of Agriculture, Kyoto University, Kyoto 606-8502, Japan; kensato@kais.kyoto-u.ac.jp

**Keywords:** chicken protein, proteases, antioxidant activity, spray-drying, freeze-drying

## Abstract

Different proteases can be applied to produce certain bioactive peptides. This study focused on the effects of some commercial proteases and drying processes on the physical, chemical, and biological properties of chicken breast hydrolysates (CBH). Chicken breast hydrolyzed with Alcalase^®^ presented a higher degree of hydrolysis (DH) than papain. Moreover, the treatment with Alcalase^®^, followed by papain (A-P), was more proficient in producing antioxidant activities than a single enzyme treatment. Conditions comprising 0.63% Alcalase^®^ (*w*/*w*) at pH 8.0 and 52.5 °C for 3 h, followed by 0.13% papain (*w*/*w*) at pH 6.0 and 37 °C for 3 h, resulted in the highest yields of DH and peptide contents. The spray-dried microencapsulated powder improved the physicochemical properties including moisture content, color measurement, solubility, and particle morphology. In summary, the dual enzyme application involving the hydrolysis of Alcalase^®^ and papain, coupled with the spray-drying process, could be used to produced antioxidant CBH.

## 1. Introduction

Food protein is essential for the body’s ability to exert certain nutritional and functional actions such as the strengthening, building, and repairing of tissues and muscle fibers [1]. Meat is a primary source of dietary protein, as it contains high amounts of essential amino acids. Chicken is the most frequently consumed meat among humans due to its affordable cost and high nutritional value. Among the common forms of ‘white meat’, chicken meat has been found to be a higher source of protein and more palatable than the meat of fish, while having no ‘fishy’ odor [2]. Thai indigenous chicken, a local genotype in Thailand, is known for its unique texture and taste. It is also associated with a high amount of protein and a low fat content when compared with broilers [3]. Furthermore, Thai indigenous chicken is rich in certain histidyl dipeptides, such as carnosine and anserine, which are known to possess significant amounts of antioxidants when compared with black-boned chickens and broilers [3]. Thus, Thai indigenous chicken could be a potential candidate for protein hydrolysate production with potentially substantial antioxidant properties.

The use of proteases in protein hydrolytic processes is an effective way to improve the physicochemical and functional properties and efficiency of protein hydrolysates. Various commercial enzymes obtained from microorganisms and plants that are employed during hydrolysate production present differing bioavailabilities depending upon their site specificity [4]. Physicochemical and functional properties, as well as the antioxidant activities of protein hydrolysates, have been correlated with enzyme specificity [5]. Alcalase^®^ (EC 3.4.21.14), a bacteria-derived protease preparation and an endopeptidase, is an alkaline serine endopeptidase that could effectively hydrolyse proteins, particularly those associated with large uncharged residues, with broad specificity [6,7]. Alcalase^®^ exhibited broad cleavage specificity and a strong hydrolysis ability in several studies involving chicken breast meat [8], animal internal organ muscles [9,10,11], and marine proteins [6,7,12,13,14]. Papain (EC 3.4.22.2), a plant-derived protease and a monothiol cysteine endoprotease, naturally exists in papaya (*Carica papaya* L.) and is mostly obtained from the latex of raw fruits. Additionally, papain has broad specificity for hydrolysing peptide bonds [15]. Papain has been used to produce bioactive peptides from various animal proteins, especially muscle protein, viz., fish meat [16,17], fowl muscle [18], and chicken breast [19]. Although papain exhibits certain non-specific actions, it prefers to cleave the peptide bond involving basic amino acids at the C-terminal of phenylalanine. Muscle protein contains myosin heavy chains that are known to possess higher amounts of lysine, arginine, and phenylalanine [16]. Thai indigenous chicken breast meat was found to be an excellent source of myosin heavy chains and actin [3]. Porcine myofibrillar protein hydrolysates obtained from papain hydrolysis that contain more hydrophobic amino acids can exhibit higher antioxidant activity than those digested with other proteases [20]. According to Sun, Pan, Guo, and Li [19], chicken breast protein digested by papain under optimal conditions exhibited significant amounts of antioxidant activities in both in vitro and in vivo tests. Moreover, poultry meat hydrolysates showed antioxidant, anti-inflammatory, and serum cholesterol-lowering properties [19,21,22]. Enzymatically modified chicken proteins may be useful in producing such beneficial natural antioxidants. These proteins can be further applied to the production of certain functional foods, pharmaceuticals, and nutraceuticals, as well as to the production of some innovative cosmeceutical products. Lobo et al. [23] reported on the significant relationship between free radicals and degenerative diseases such as aging, atherosclerosis, and osteoporosis. Thus, natural antioxidants could be applied to alleviate this disorder.

Functional and physical properties of protein hydrolysate products are influenced by different drying methods [24]. The spray-drying process is known to have an influence on hydrolysate powder and could ultimately increase antioxidant properties, product recovery, and dry efficiency [8]. The inhibition of DPPH radical formation by chicken breast protein hydrolysates varied from 38.7% to 59.4% depending on the inlet air temperature used in the spray-drying process [8]. However, some research groups have reported that the drying process for egg white protein hydrolysates had no influence on antioxidant activity [25]. According to those reports, the efficiency of the drying process on the biological and physicochemical properties of chicken breast protein hydrolysate (CBH) was rare and unclear, particularly with regard to this Thai indigenous chicken breed.

Therefore, the objective of this study was to determine the effects of a combination of hydrolytic enzyme treatments and drying processes (spray- and freeze-drying) on the physiochemical and antioxidant properties of CBH. Accordingly, commercial Alcalase^®^ and papain were selected to determine the extent of hydrolysis in single- and dual-enzymatic treatments.

## 2. Materials and Methods

### 2.1. Chemicals

Alcalase^®^ (3.018 U/mL) and papain (30,000 USP-U/mg) were provided from Merck (Darmstadt, Germany) were produced from *Bacillus licheniformis* and papaya (Carica papaya), respectively. Maltodextrin (DE10; CP Kelco, Lille Skensved, Denmark) was used as a wall material. O-phthalaldehyde (OPA) was purchased from Merck (Tokyo, Japan). L-serine was acquired from Merck (Burlington, MA, USA). Bovine serum albumin (BSA) was purchased from Thermo Fisher Scientific (Meridium, Waltham, MA, USA). 2,2-Diphenyl-1-picrylhydrazyl (DPPH), 2,2′-Azinobis-(3-ethylbenzothiazoline-6-sulfonic acid) diammonium salt (ABTS), 2,4,6-Tris(2-pyridyl)-s-triazine (TPTZ), L-glutathione reduced (GSH), and 6-hydroxy-2,5,7,8-tetramethylchroman-2-carboxylic acid (Trolox) were provided by Sigma-Aldrich (Louis, MO, USA). Other chemicals used in this study were of analytical grade.

### 2.2. Preparation of Chicken Breast Meat

Whole breasts of Thai indigenous chickens were obtained from a commercial farm in Chiang Mai, Thailand. Thai indigenous chickens were slaughtered at 4 months with approximate body weights of 1.3 to 1.4 kg. Breast meat used in the study was 24 h postmortem. Visible subcutaneous fat and connective tissues were trimmed and removed. Each sample was rinsed using distilled water and minced to a homogenate with a blender (DPA130, TEFAL, Groupe SEB, Lyon, France). The minced meat was immediately frozen at −20 °C until analysis and is referred to as chicken meat homogenate.

### 2.3. Enzymatic Hydrolysis of Chicken Breast Meat

To study the effect of either Alcalase^®^ or papain on hydrolysis capacities, the minced meat was added to 0.1 M sodium phosphate buffer pH 8.0 for Alcalase^®^ or pH 6.0 for papain in a ratio of 1 to 5 (*w/v*) and homogenized using a homogenizer (Nissei AM-8 homogenizer; Nissei Corporation, Tokyo, Japan). The homogenate was incubated with various concentrations of Alcalase^®^, 0.31, 0.63, 0.94, and 1.25% (*w*/*w*), at 52.5 °C for 3 h, and was labelled CBH:A. The hydrolysis with papain used various concentrations of papain, 0.06, 0.13, 0.25, and 0.50% (*w*/*w*), at 37 °C for 3 h. The homogenate with papain was labelled CBH:P. To inactivate proteases, the reaction mixture was boiled 10 min and cooled down to ambient temperature. The supernatant was separated by centrifugation (MX-305; Tomy Seiko, Tokyo, Japan) at 10,000× *g* for 10 min and then filtered using filter papers (Whatman No. 1; Merck KGaA, Darmstadt, Germany). The filtrate was lyophilized and stored at −20 °C for further use.

To study the effect of dual enzyme treatment, Alcalase^®^ and papain were used at the optimal conditions derived from the previous step. Concentrations of 0.63% (*w*/*w*) Alcalase^®^ at 52.5 °C for 3 h and 0.13% (*w*/*w*) papain at 37 °C for 3 h were applied to chicken meat homogenate. The first protocol was designed to involve hydrolysis with Alcalase^®^ followed by papain, denoted as A-P. After hydrolysis by Alcalase^®^, the hydrolysate was inactivated by adjusting the pH to 6.0. Papain was then added, and the specimen was further incubated under the condition as described above to obtain hydrolysates CBH:A-P. The second protocol set the reaction with papain followed by Alcalase^®^, labelled as P-A. The hydrolysate was inactivated by adjusting the pH to 8.0 after digestion by papain. Alcalase^®^ was then added, and the specimen was further incubated under conditions as described above to obtain hydrolysates CBH:P-A.

### 2.4. Degree of Hydrolysis

The degree of hydrolysis (DH) was used to evaluated peptide content that was produced during protein digestion using the method of Pedroche et al. [26] and was calculated as percent hydrolysis (%, DH) using the following formula:

DH (%) = [Peptides in hydrolysate/Total peptides in raw material] × 100

Peptides in hydrolysate and total peptide content in raw material were measured by the o-phthaldialdehyde (OPA) method described by Nielsen and groups [27]. Briefly, 4 mL of 6 N HCl was added to 1 mL of hydrolysate, and then the mixture was heated at 110 °C for 18 h. Subsequently, the mixture was adjusted to neutral with 6 N NaOH and then diluted with distilled water. One mL of OPA reagent was added to 50 μL hydrolysate and mixed thoroughly. After incubating at ambient temperature for 2 min, the mixture was analyzed by measuring its optical density at a wavelength of 340 nm. The peptide content of hydrolysate sample was calculated using the L-serine standard curve. The value is expressed in units of mg L-serine per mL sample.

### 2.5. Preparation of Microencapsulated CBH Powder

To prepare the feed mixture, maltodextrin was added directly to the CBH prepared at the optimum conditions (A-P: 0.63% (*w*/*w*) Alcalase^®^ at 52.5 °C for 3 h, followed by 0.13% (*w*/*w*) papain at 37 °C for 3 h), and the mixture was stirred until it reached 15° Brix (RHB-20 ATC, JEDTO; Prononics Co., Ltd., Pathumthani, Thailand) of the final solid content. The feed mixture was fed into the chamber of a spray dryer (JCM Engineering Concept Co., Ltd., Bangkok, Thailand) at a feed flow rate of 60 mL/min through a nozzle atomizer under an intake air temperature of 190 ± 10 °C. The spray-dried CBH powder was immediately collected and stored in a metallized bag after drying. For the freeze-drying process, the dispersion was frozen for 48 h before being placed in a freeze dryer (Labconco, Kansas City, MO, USA) and subsequently dried at −43.2 °C under a pressure of 0.09 mbar for 24 h. The lyophilized chicken breast protein hydrolysate powder was collected and then treated as in the previous step.

### 2.6. Physical and Chemical Properties of the Microencapsulated CBH Powder

The moisture content of the hydrolysate sample was calculated from the weight loss after heating the sample in a drying oven (Memmert GmbH, Schwabach, Germany) at 105 °C according to Association of Official Agricultural Chemists (AOAC) [28].

The color of the hydrolysate sample was determined using a spectrophotometer (CR-400; Konica Minolta, Tokyo, Japan), according to the CIELAB (L*, a*, b*) system, where L* indicates lightness (0 = black and 100 = white), and a* and b* are coordinates for green (−a*)/red (+a*) and blue (−b*)/yellow (+b*).

The morphology of the hydrolysate sample was obtained using a scanning electron microscope (SEM, JSM-5200; JEOL Ltd., Tokyo, Japan). The powder was applied on the surface of a sticker on a specimen holder. Then, the sample was coated with 99% pure gold using JFC-1100E Auto fine coater, before being analyzed using an SEM.

The solubility of the hydrolysate sample was determined according to the method of Shittu and Lawal [29] with a slight modification. The sample was dispersed in distilled water. The solution was stirred for 30 min by a magnetic stirrer at ambient temperature and then centrifuged at 6000 rpm for 20 min. The supernatant was dried at 105 °C for 24 h. The solubility percentage was reported from the weight of dried solid.

### 2.7. Antioxidant Activities

#### 2.7.1. Scavenging Activity on DPPH Free Radicals

The antioxidant activity of CBH was measured by its DPPH free radical trapping ability according to a modified method of Alam et al. [30]. The hydrolysate solution was prepared by dissolution in distilled water at a concentration of 10 mg/mL (*w/v*). This solution was mixed with 0.2 mM fresh DPPH in methanol. The absorbance of the reaction mixture was investigated at a wavelength of 517 nm after incubation in the dark for 30 min. Trolox was used as a standard, while L-glutathione was a positive control. The results were expressed as percent inhibition and were compared to the control.

#### 2.7.2. Scavenging Activity on ABTS^•+^ Cation

The scavenging activity of ABTS^•+^ of CBH was investigated according to Alam, Bristi, and Rafiquzzaman [30], with a slight modification. ABTS^•+^ was prepared by the reaction between 7 mM ABTS in water and 2.45 mM potassium persulfate (1:1) and was stored in the dark at ambient temperature for 12–16 h before use. CBH solution (10 mg/mL) and standard glutathione were added to a working ABTS^•+^ solution, and the mixture was incubated for 10 min. The optical density of the final product was measured at 734 nm. The data were standardized using Trolox and are presented as percent inhibition of ABTS^•+^ radical formation.

#### 2.7.3. Ferric Reducing Antioxidant Power

The ferric reducing antioxidant power (FRAP) of the sample was investigated using FRAP assay, as described by Benzie and Strain [31], with a slight modification. The active reagent was prepared by mixing 300 mM sodium acetate buffer, 10 mM TPTZ, and 20 mM ferric chloride in the proportions 10:1:1 at 37 °C. The fresh reagent was mixed with the sample, and the mixture was kept in the dark. After 30 min., this reaction was monitored by measuring the change in absorbance at 593 nm. Trolox was used as a standard. The FRAP values were obtained by comparing the absorbance change in the test mixture with a standard curve and the results expressed as µM Trolox equivalent per gram sample.

### 2.8. Statistical Analysis

The results were analyzed and expressed as mean ± standard deviation, using the Statistical Package for the Social Sciences (SPSS^®^; SPSS Inc., Chicago, IL, USA) version 23.0 software. Analysis of variance (ANOVA) was used to determine significant differences (*p* < 0.05) among the samples. The difference between means was detected using Duncan’s multiple range test (DMRT). For a drying method comparison, a *t*-test was also performed using SPSS^®^.

## 3. Results

### 3.1. Effect of Alcalase^®^ and Papain on Hydrolysis of Chicken Breast Meat

**Figure 1:**
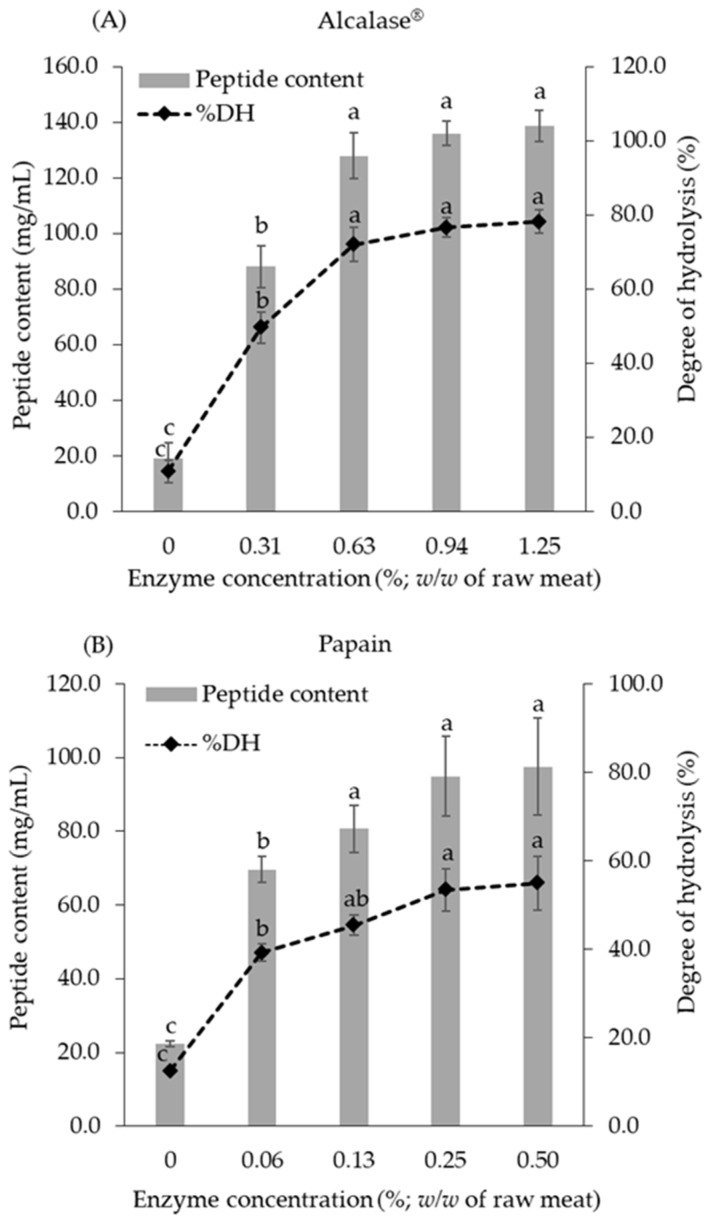
Effects of enzyme concentration on degree of hydrolysis (DH) and peptide content in chicken meat hydrolysates. (**A**): Alcalase^®^ and (**B**): papain. Different letters in the bar graph indicate significant differences at *p* < 0.05.

The effects of various concentrations of Alcalase^®^ and papain on DH and peptide content are shown in Figure 1. The results indicate that the ranges of enzyme concentrations of both Alcalase^®^ and papain were directly related to DH and peptide content. The maximum degrees of efficacy for CBH:A and CBH:P were 0.63 and 0.13% (*w*/*w*), which then produced DH values of 70.1 ± 7.8 and 45.4 ± 5.3%, respectively. In accordance with DH, the peptide content was 97.20 and 56.66 mg/mL for CBH:A and CBH:P, respectively. Therefore, the above-mentioned conditions can be considered the optimal hydrolysis conditions to provide maximum DH and peptide content.

### 3.2. Effect of Sequential Dual Protease Treatment on Chemical and Antioxidant Properties of CBH

Alcalase^®^ and papain at optimal concentrations of 0.63% and 0.13%, respectively, were selected for further studies focusing on their hydrolytic efficiency and antioxidant activity. The digestive conditions involving sequential dual enzymes (either A-P or P-A) presented greater DH and peptide content than the conditions employing a single enzyme, as is shown in Table 1. CBH:A-P was associated with the highest DH content (74.47%). However, in this study, the use of sequential dual enzymes, namely CBH:P-A, produced lower DH (54.75%) than did the single enzyme (CBH:A, 62.99%). Notably, the lowest DH content was observed for CBH:P (31.92%).

For the CBH produced through a series of processes involving sequential dual enzyme hydrolysis, CBH:A-P displayed the highest antioxidant potential, followed by CBH:P, CBH:P-A, and CBH:A (Table 1). However, CBH:A was more significantly (*p* < 0.05) involved with the inhibition of the DPPH radical (13.83 ± 1.21%) than was CBH:P-A (11.25 ± 2.30%), but it did not differ significantly (*p* > 0.05) in terms of the inhibition of the ABTS radical (38.73 ± 1.48% and 34.88 ± 0.21%, respectively). Furthermore, DPPH and ABTS radical scavenging activities of CBH:A-P were found to be greater than those of CBH:A.

### 3.3. Effect of Drying Process on Physicochemical Properties of CBH Powder

The sequential dual enzyme digestion of Alcalase^®^, followed by the Papain (A-P) treatment, was effective for chicken hydrolysate production, as can be observed by the maximum antioxidant capacity. Thus, CBH:A-P was used to study the effect of the drying processes, namely spray-drying and freeze-drying, on physicochemical properties. The physical and chemical properties of CBH:A-P are shown in Table 2. CBH:A-P obtained by either spray-drying or freeze-drying with maltodextrin were significantly different in color (*p* < 0.05). The color of the spray-dried powder was darker than that obtained from the freeze-drying process, while the degree of redness and yellowness (lower a* and b* values) resulting from the freeze-drying process was less than that which resulted from spray-drying. CBH:A-P produced by the spray-drying method exhibited the highest degree of powder solubility (92.5 ± 1.6%) when compared with that of the CBH:A-P produced by freeze-drying. However, the greatest moisture content was observed from the freeze-drying method (3.09 ± 0.4%). Furthermore, it was intriguing that in this study, the peptide content of the CBH powder was not significantly different when both drying methods were compared. Even though there were differences in physicochemical characteristics, similarities of antioxidant activities of CBH:A-P from both drying processes were observed, as is shown in Table 3.

**Figure 2:**
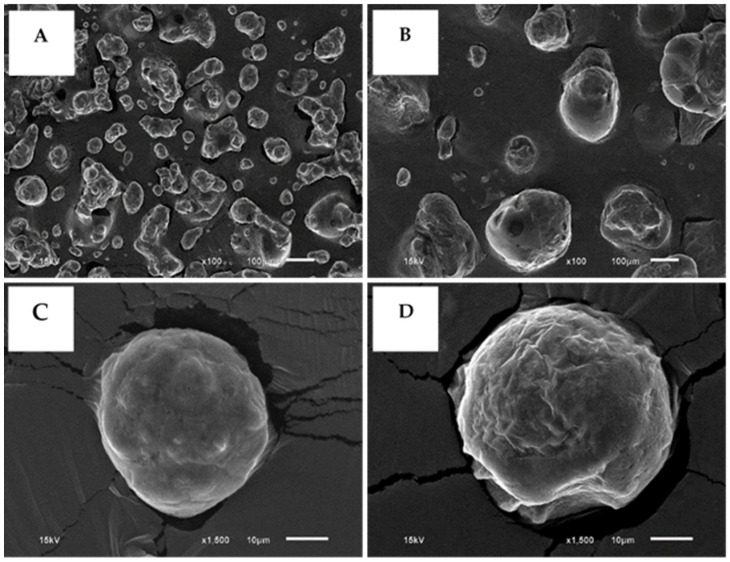
Micrographs of chicken breast hydrolysate powder produced by Alcalase^®^ and followed by Papain (CBH:A-P) that were dried with spray-drying and freeze-drying using scanning electron microscopy (SEM). Spray drying at 100× (**A**) and 1500× (**C**). Freeze-drying at 100× (**B**) and 1500× (**D**).

The structural analysis of chicken hydrolysate powder (CBH:A-P) obtained from the spray-drying and freeze-drying processes was investigated using SEM (Figure 2). The images revealed clear differences in particle morphology and size distribution. The samples obtained from spray-drying (Figure 2A) were smaller in size than those obtained from freeze-drying (Figure 2B). Importantly, the spray-dried powder presented a spherical shape with a formation of link bridges and a rough surface, while the freeze-dried powder presented an indefinite structure and a shriveled surface (Figure 2C,D).

## 4. Discussion

Proteases can produce certain peptide sequences that are responsible for different bioactivities. DH has been applied as an indicator for the cleavage of the peptide bond in a hydrolytic reaction. This was also found to have been influenced by the enzyme reaction rate, enzyme specificity, and substrate affinity [27]. In this study, the hydrolytic efficiency of Alcalase^®^ and papain indicated by the DH and peptide content were 6.5- and 3.6-fold higher than those of the control. According to their enzyme specific activities, Alcalase^®^ was found to be more potent for protease activity than papain in the chicken hydrolysate preparation. This outcome was in line with the results of other studies, which reported that Alcalase^®^ could provide greater DH content for bovine lung protein hydrolysis [11], porcine liver [9,10], and sliver carp muscles [14] when compared to papain and other proteases. Alcalase^®^ exhibits broad specificity and is capable of generating peptides containing hydrophobic amino acids [8]. However, papain could effectively cleave the peptide bonds of basic amino acids, leucine, or glycine, while also hydrolyzing the ester and amide bonds [15]. The data suggest that the DH and peptide properties were dependent upon enzyme specificity.

Treatment with dual sequential enzymes could enhance the cleavage of large peptides from hydrolysates, while smaller peptides were obtained when compared to the treatment in which only a single enzyme was used [32,33]. The results from this study were consistent with those of Li, Yu, Goktepe, and Ahmedna [33], all of which indicated that the sequential treatment provided significant proteolytic capacities. The DH of CBH:P was determined to be lowest, while this value may support lower %DH of CBH:P-A than CBH:A-P due to a broader degree of cleavage specificity and the stronger hydrolysis ability of Alcalase^®^ [34]. It is likely that papain could specifically cleave the peptide bonds that may have been contributed by the adjacent lysine, arginine, and phenylalanine [16].

Due to differences in the protein cleavage sites, sequential A-P or P-A enzyme treatments of chicken breast proteins were investigated. Some complementary effects on antioxidant activity were expected. DPPH, ABTS, and FRAP values corresponded with those of the DH of CBH with higher DH values corresponding to higher antioxidant properties. However, CBH:P exhibited the lowest DH but not the lowest values for DPPH and ABTS. Apart from the DH, the antioxidant activities of protein hydrolysates obtained from various conditions and proteases were found to be different due to the involvement of different types of enzymes and the differing molecular weights of the peptides. This may also have been due to the existing diversity of peptides, amino acid compositions, or sequences [35]. The antioxidant activity presented by CPH might have possibly been due to the hydrophobic amino acid content [36]. In addition, the presence of hydrophobic amino acid at the C-terminal position could also enhance the scavenging activity [37]. Thus, further investigations of these parameters in the resulting CBH samples will be needed in the future.

Different drying methods had significant impacts on the physicochemical properties of CBH:A-P. Lower L* values, along with higher a* and b* values that are associated with spray-drying, were influenced by the higher temperatures employed when drying. Both drying methods exhibited a negative a* value indicating a greenish hue. Remarkably, the b* value was higher in the spray-dried powder, which caused significant changes in color and had a brown appearance. The high temperatures induced by the Mallard reaction between the carbonyl groups of reducing sugars and the primary amino groups of amino acids [38] led to the generation of brown-colored compounds. The higher solubility value of CBH:A-P that had been dried by spray-drying may be related to the resulting smaller particle size. This is suggested because smaller particles would likely have increased particle surface area, leading to greater solubility [24]. The large particle size obtained from the freeze-drying process might have been attributed to the low temperature and low breaking force applied on the frozen liquid, which could have converted the liquid into droplets and alter their topological surface area [25]. Moreover, the spray-drying technique employed a higher air temperature than the freeze-drying process and could more effectively remove the wetted surface of the spray-dried powder. For this reason, our results indicated that a higher moisture content occurred from the freeze-drying technique. Additionally, rapid freezing temperatures that were lower than −40 °C lessened the pore size of the outer layer, which tends to act as a barrier for sublimation [39]. This could explain the higher moisture content in CBH:A-P that had been freeze-dried.

Several of the link bridges present in the spray-drying treatment could have been caused by hygroscopicity. Kurozawa, Park, and Hubinger [21] reported that the protein hydrolysates of chicken meat were found to contain these links between particles as a consequence of their higher degree of hygroscopicity. According to Saikia et al. [40], the higher hygroscopicity values associated with the spray-dried powder were also related to the smaller particle size and reduced moisture content when compared to the freeze-dried powder. Additionally, the different surface areas of the powders might have been due to the drying temperatures that were employed, as has been discussed in Tonon et al. [41]. The production of powder by low temperatures led to a shriveled surface on the particles, while increased temperatures resulted in a larger number of smooth-surfaced particles [41]. In addition, the surface of the protein hydrolysate was found to often collapse when using the freeze-drying method [42]. However, Kurozawa, Park, and Hubinger [21] reported that the rough surface associated with the spray-drying process can usually be attributed to slow film formation during the drying and cooling phases of the process.

Kurozawa, Park, and Hubinger [8] reported that the DPPH inhibition activity of chicken meat protein hydrolysates varied based on the temperatures used in drying process. Notwithstanding, no significant differences were observed in terms of DPPH inhibition activity, ferric reducing ability, and lipid peroxidation inhibitory activity of egg white protein hydrolysates before and after being dried [25]. This outcome agrees with the ultimate findings of our study. As has been mentioned above, the characterization of peptides derived from both drying processes needs to be further investigated to clarity the effects of antioxidant activities.

## 5. Conclusions

CBH produced by a series of dual enzyme hydrolysis provides better physical-biochemical properties than that which is produced by single protease hydrolysis. Peptides obtained from Alcalase^®^ following the Papain (A-P) treatment with known physicochemical characteristics can yield potential antioxidant activities that proficiently scavenged free radicals and reduced ferric ion. Spray-drying was found to be more efficient than freeze-drying for microencapsulation in terms of solubility. However, a better color of the hydrolysate was derived through freeze-drying. The results from this study suggest that CBH could be applied as a natural antioxidant in the food industry. Moreover, the outcomes of this investigation can offer insights into the possibility of effective applications of these hydrolysates in the development of food ingredients and functional foods, as well as in a number of other related fields in the future. However, there is a need to further study the characterization of antioxidant peptides with regard to their structure–antioxidant relationship, amino acid profiles, amino acid sequences, and sensory attributes, as well as the shelf-life of the resulting CBH.

## Figures and Tables

**Table 1:** Degree of hydrolysis (DH), peptide content, and antioxidant activity of chicken breast hydrolysates (CBH) under various conditions ^1,2^.

Samples	DH (%)	Peptide Content (mg/mL)	FRAP Value (µM Trolox Equivalent/g Sample)	Inhibition (%, 10 mg/mL)	
				DPPH Assay	ABTS Assay
CBH:A 0.63%	62.99 ± 2.62 ^b^	111.82 ± 4.65 ^b^	0.41 ± 0.02 ^d^	13.83 ± 1.21 ^c^	34.88 ± 0.21 ^b^
CBH-P 0.13%	31.92 ± 3.27 ^d^	56.66 ± 5.80 ^d^	0.54 ± 0.03 ^b^	19.55 ± 0.32 ^b^	41.59 ± 5.95 ^a^
CBH:A-P	74.47 ± 1.78 ^a^	133.77 ± 3.15 ^a^	0.71 ± 0.02 ^a^	28.32 ± 0.44 ^a^	48.97 ± 1.93 ^a^
CBH:P-A	54.75 ± 1.76 ^c^	97.20 ± 3.13 ^c^	0.54 ± 0.03 ^c^	11.25 ± 2.30 ^d^	38.73 ± 1.48 ^b^
GSH	-	-	368 ± 14.06	44.27 ± 0.69	38.86 ± 1.56

Different letters in the same column indicated significant differences at *p* < 0.05. ^1^ Values are expressed as mean ± standard deviation. ^2^ CBH refers to chicken protein hydrolysates; CBH:A, CBH:P, CBH:A-P, and CBH:P-A refer to the CHB produced by hydrolysis with Alcalase^®^, papain, Alcalase^®^ followed by Papain, and Papain followed by Alcalase^®^, respectively; GSH refers to L-glutathione at 0.1 mg/mL.

**Table 2:** Physical and chemical properties of chicken breast hydrolysate powder produced by Alcalase^®^ and followed by Papain (CBH:A-P) under different drying conditions ^1^.

Powder Properties	Drying Conditions	
	Spray-Drying	Freeze-Drying
Color parameters		
L*	88.50 ± 0.06 ^b^	91.20 ± 0.02 ^a^
a*	−7.45 ± 0.07 ^a^	−8.12 ± 0.01 ^b^
b*	16.14 ± 0.03 ^a^	14.27 ± 0.20 ^b^
Solubility (%)	92.50 ± 1.65 ^a^	87.76 ± 1.09 ^b^
Moisture (%)	2.15 ± 0.24 ^b^	3.09 ± 0.44 ^a^
Peptide content (*w*/*w*)	0.59 ± 0.01 ^b^	0.63 ± 0.01 ^a^

Different letters in the same row indicated significantly difference at *p* < 0.05. ^1^ Values are expressed as mean ± standard deviation.

**Table 3:** Antioxidant activities of the chicken breast hydrolysate powder produced by Alcalase^®^ and followed by Papain (CBH:A-P) under different drying conditions ^1,2^.

Drying Conditions	FRAP Value (µM Trolox Equivalent/g Sample)	ABTS Inhibition Activity (%) (10 mg/mL)
Spray-drying	0.28 ± 0.06 ^a^	57.50 ± 0.87 ^a^
Freeze-drying	0.21 ± 0.08 ^a^	53.68 ± 3.38 ^a^
GSH	368 ± 14.06	38.86 ± 1.56

Different letters in the same row indicated significantly difference at *p* < 0.05. ^1^ Values are expressed as mean ± standard deviation at *p* < 0.05. ^2^ GSH refers to L-glutathione at 0.1 mg/mL.

## Data Availability

The authors declare that all of the data and the material used in this study are available within this article. All data generated or analyzed in this study can be obtained from corresponding author upon reasonable request.
